# Hospital Week at Northampton

**Published:** 1894-02-03

**Authors:** 


### Editor's Letter-Box.

### HOSPITAL WEEK AT NORTHAMPTON.

The Secretary of this fund writes: "I find on page 272 of The Hospital of January 20th a paragraph referring to the Northampton Hospital Week Fund, in which it says, 'Committee has handed over to the funds of that institution the sum of £200.' This should be £900. Please rectify in your next issue."

